# Pre-sterilization of vancomycin-loaded cement spacers: impact on antibacterial efficacy against *Staphylococcus aureus*

**DOI:** 10.1007/s00264-026-06829-9

**Published:** 2026-05-19

**Authors:** Kulapat Chulsomlee, Pisut Pongchaikul, Rungroj Sadchaphaiboonkit, Jinnipa Arunothai, Paninee Mongkolsuk, Nithid Sri-utenchai

**Affiliations:** Chakri Naruebodindra Medical Institute, Faculty of Medicine Ramathibodi Hospital, Mahidol University, Bangkok, Thailand

**Keywords:** Antibiotic-loaded bone cement, Vancomycin, Formaldehyde sterilization, Periprosthetic joint infection, Polymethylmethacrylate (PMMA)

## Abstract

**Background:**

Antibiotic-loaded polymethylmethacrylate (PMMA) cement spacers are widely used in periprosthetic joint infection and chronic osteomyelitis. Manual prefabrication and sterilisation of non-commercial antibiotic-loaded cement may reduce operative time and cost; however, the effects of sterilisation and storage on antimicrobial efficacy remain unclear.

**Methods:**

Manually prefabricated PMMA cement containing vancomycin (2 g or 4 g) underwent formaldehyde gas sterilisation (FO) and storage for one, four or seven days. Antibiotic elution was evaluated over 28 days. Antimicrobial activity against *Staphylococcus aureus* ATCC 25923 was assessed using minimum inhibitory dilution (MID) testing at predefined time points. Given the small number of specimens per subgroup, all comparisons should be interpreted as preliminary and hypothesis-generating.

**Results:**

FO sterilisation significantly reduced antimicrobial activity during the early elution phase. In the 4—g vancomycin group, Day 1 MID values were significantly higher in one day storage sterilised specimens than in specimens stored for four or seven days (1024 µg/mL vs. 213 µg/mL and 213 µg/mL, respectively; *P* < 0.001). Differences persisted at early time points but were no longer significant during the sustained elution phase (Days 14–28; *P* > 0.05). Overall, sterilised cement containing 4 g of vancomycin demonstrated higher MID values than 2 g cement during the early and mid-elution phases (Days 1–14; *P* < 0.01). MID values in all sterilised specimens remained several-fold above inhibitory thresholds for *S. aureus* throughout the 28-day period.

**Conclusions:**

FO sterilisation transiently reduces vancomycin antimicrobial activity during the early elution phase (Days 1–7) but does not affect sustained antimicrobial efficacy compared with non-sterilised cement. Based on these findings, vancomycin-loaded PMMA cement containing 4 g of antibiotic may be sterilised and stored for up to seven days while maintaining MIC values several-fold above inhibitory thresholds for *Staphylococcus aureus* throughout the 28-day elution period.

## Introduction

Periprosthetic joint infection (PJI) is a serious complication that commonly arises after total knee or hip arthroplasty. The incidence of PJI has been reported in approximately 1–2% of primary arthroplasties, increasing to 3–5% in revision procedures [1]. For chronic infection, the current gold standard remains a two-stage revision: first, explantation of the infected prosthesis with extensive surgical debridement, followed by implantation of an antibiotic-loaded cement spacer (ACS), and later, after infection control, reimplantation of a new prosthesis [2, 3].

In current practice, ACS fabrication is performed intraoperatively by mixing polymethyl methacrylate bone cement (PMMA) with 2–4 g of antibiotic and molding it to the shape of the removed prosthesis. This process typically prolongs operative time by 20–30 min [3]. Prolonged surgical duration has consistently been associated with increased intraoperative blood loss, higher transfusion rates, and a greater risk of postoperative complications, including surgical site infection and venous thromboembolism [4, 5]. To reduce operative time, the concept of prefabricating ACSs before surgery and sterilizing them through the hospital’s standard processes has been proposed [6, 7].

However, limited data exist on the impact of sterilisation on the antimicrobial properties of vancomycin-loaded PMMA spacers. The objective of this study is to evaluate the effect of standard sterilisation procedures on the antibiotic performance of pre-formed, vancomycin-loaded bone cement spacers, specifically examining minimum inhibitory dilution (MID)-based antimicrobial activity, a functional bioassay approach that quantifies the antimicrobial potency of cement eluate rather than the susceptibility of the test organism.

## Materials and methods

### Specimen preparation

Antibiotic-loaded bone cement specimens were prepared using polymethyl methacrylate (PMMA; Palacos®). Vancomycin powder was incorporated into the PMMA powder at two concentrations: 2 g and 4 g per cement batch. The liquid monomer was then added, and mixing was performed according to the manufacturer’s instructions.

During the dough phase, the cement mixture was packed into standardised metal molds to ensure consistent specimen size and shape. After full polymerisation, the specimens were removed from the molds and allowed to cool to room temperature. Each specimen was weighed, and the baseline weight was recorded.

### Sterilisation protocol

Specimens assigned to the sterilisation group underwent formaldehyde gas sterilisation at the Central Supply Sterilisation Unit (CSSD), Chakri Naruebodindra Medical Institute (CNMI). Sterilisation was performed using formaldehyde gas at 60–80 °C, following the CSSD’s standard operating procedures.

After sterilisation, specimens were placed and maintained in a closed, sealed sterile package according to the hospital’s standard sterile storage protocols within the CSSD. Non-sterile specimens were stored under the same environmental conditions without undergoing sterilisation.

### Grouping

**Fig. 1:**
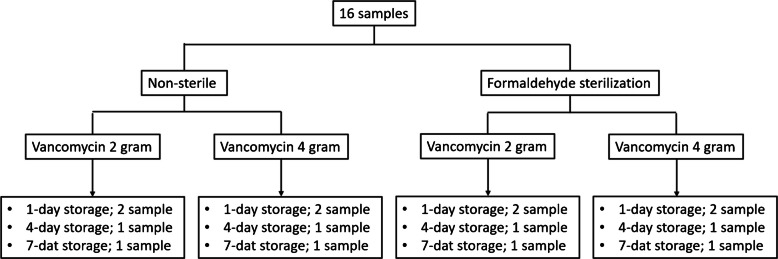
Flow diagram showing preparation and allocation of vancomycin-loaded PMMA cement specimens. Sixteen specimens (2 g or 4 g vancomycin) were divided into sterilised and non-sterilised groups and further assigned to storage durations of 1, 4, or 7 days prior to analysis

A total of 16 cement specimens were fabricated—eight specimens containing 2 g of vancomycin and 8 containing 4 g. Each concentration group was divided equally into a non-sterile control group and a sterilisation group. Specimens in the sterilisation group underwent standard sterilisation, while non-sterile specimens were kept as controls. To evaluate the impact of storage duration following sterilisation, specimens were further assigned to three time-interval subgroups (Fig. 1):

1. **one day storage:** four specimens (two sterile, two non-sterile)
2. **four day storage:** two specimens (one sterile, one non-sterile)
3. **seven day storage:** two specimens (one sterile, one non-sterile)

This distribution was applied separately to both the 2—g and 4—g vancomycin groups. After fabrication, the weight of each cement spacer specimen was measured and recorded prior to further analysis. The four day and seven day storage subgroups each contained only a single sterilised specimen; statistical comparisons within these subgroups therefore have limited precision and should be interpreted with caution.

#### Antibiotic elution testing and antimicrobial susceptibility assay

**Fig. 2:**
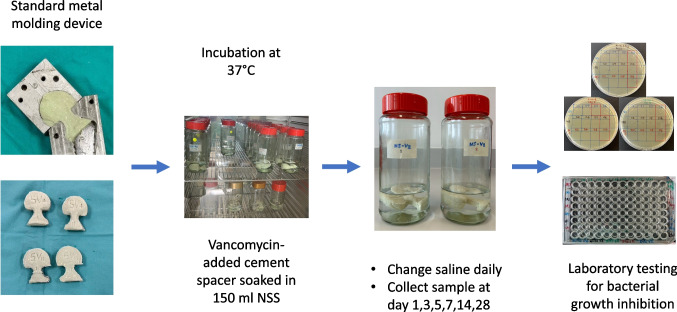
Schematic of PMMA cement preparation and elution testing for MID analysis against *Staphylococcus aureus*

Each specimen was immersed in 150 mL of saline (0.9% sodium chloride solution) and incubated at 37 °C. The solution was replaced daily: specimens were removed, rinsed with sterile saline, and transferred to fresh containers containing 150 mL of new saline. Eluate samples were collected on Days one, three, five, seven, 14, 21, and 28, then stored at –80 °C until analysis (Fig. 2).

Antimicrobial potency of the eluent was determined based on broth microdilution. *Staphylococcus aureus* ATCC 25923 was cultured in cation-adjusted Mueller–Hinton broth (CAMHB) and adjusted to a 0.5 McFarland standard (approximately 1.5 × 10^8^ CFU/mL). A two-fold serial dilution of each eluate sample was prepared in CAMHB, and equal volumes of diluted eluate and bacterial inoculum were added to each well to achieve a final bacterial concentration of 5 × 10^5^ CFU/mL. At each elution time point, each eluate was tested in triplicate to minimise technical variability. Microtiter plates were incubated at 37 °C for 16—20 h, after which bacterial growth was assessed visually.

The antimicrobial potency of each eluate was expressed as the minimum inhibitory dilution (MID), which is defined as the highest eluate dilution and expressed as the equivalent vancomycin concentration (µg/mL), that completely inhibited visible bacterial growth. Higher MID values indicate greater antimicrobial activity in the eluate. Critically, MID is a functional bioassay metric that quantifies the antimicrobial potency of the cement eluate matrix, and is conceptually distinct from conventional minimum inhibitory concentration (MIC) testing, in which a single antibiotic is tested against an organism at defined concentrations to determine susceptibility. Rather than characterising organism susceptibility, MID characterises eluate activity: it represents how far a collected eluate sample can be diluted before it loses its ability to inhibit bacterial growth. Serial dilution-based functional bioassay has been used in prior PMMA elution research to assess active antibiotic content in eluate, including studies by Meeker et al*.* [8] and Shaw et al. [9], where eluate activity was compared against MIC breakpoints to confirm antimicrobial preservation. A MID value of ≥ 2 µg/mL (equivalent vancomycin concentration) indicates that the eluate retains activity above the CLSI susceptibility breakpoint for *S. aureus*. In vitro experiments involving bacteria and antibiotic were approved by the Institutional Biosafety Committee (IBC MURA 2026/1).

### Statistical analysis

A mixed-effects model was used to compare MID values between groups while accounting for repeated measurements over time. This model was selected to accommodate the unbalanced group sizes arising from the study design, in which the 1-day storage subgroup contributed two replicate specimens per condition whereas the four day and seven day subgroups each contributed one. Mean differences with 95% confidence intervals were calculated, and a two-sided P value < 0.05 was considered statistically significant. Given the small number of specimens per subgroup, particularly in the four day and seven day storage conditions, all within-subgroup comparisons should be considered exploratory; the resulting confidence intervals are wide, and the findings require confirmation in studies with larger sample sizes. Analyses were performed using Stata software, version 18 (College Station, TX: StataCorp LLC).

## Results

### Vancomycin 2 g group

Across all storage durations (Table 1), Day 1 eluates demonstrated a significant reduction in antibacterial activity against *Staphylococcus aureus* in sterilised specimens compared with non-sterilised controls (*p* < 0.05). The magnitude of reduction increased with longer storage duration. Specimens stored for one day showed the smallest decrease in MID (mean difference − 42.67; 95% CI − 71.67 to − 13.66; *p* = 0.004), whereas specimens stored for seven days exhibited the greatest reduction (mean difference − 384; 95% CI − 395.17 to − 372.83; *p* < 0.001).

**Table 1:** Minimum inhibitory dilution (MID) of vancomycin eluted from polymethylmethacrylate bone cement containing 2 g of vancomycin, comparing non-sterilised and formaldehyde gas-sterilised specimens across storage durations

Outcome	MID (սg/mL)		Mean difference	*P*-value
	mean ± SD or	mean ± SD or	(95%CI)	
*NS-V2 VS S-V2*	LS mean (95%CI)	LS mean (95%CI)		
Day 1	298.67 ± 104.51	256 ± 0	−42.67 (−71.67,−13.66)	0.004*
Day 3	32 ± 0	32 ± 0	1.14e-13 (−29.01,29.01)	1.000
Day 5	24 ± 8.76	16 ± 0	−8 (−37.01,21.01)	0.589
Day 7	13.33 ± 4.13	12 ± 4.38	−1.33 (−30.34,27.67)	0.928
Day 14	8 ± 0	8 ± 0	5.68e-14 (−29.01,29.01)	1.000
Day 21	5.33 ± 2.07	4 ± 0	−1.33 (−30.34,27.67)	0.928
Day 28	4 ± 0	4 ± 0	5.68e-14 (−29.01,29.01)	1.000
*NS-V2 (4) VS S-V2 (4*)	**NS-V2(4) group**	**S-V2 (4) group**		
Day 1	341.3 ± 147.80	128 ± 0	−213.33 (−265.35,−161.32)	< 0.001*
Day 3	64 ± 0	42.67 ± 18.48	−21.33 (−73.35,30.68)	0.421
Day 5	16 ± 0	32 ± 0	16 (−36.02,68.02)	0.547
Day 7	16 ± 0	32 ± 0	16 (−36.02,68.02)	0.547
Day 14	16 ± 0	16 ± 0	−1.14e-13 (−52.02,52.02)	1.000
Day 21	8 ± 0	8 ± 0	−1.14e-13 (−52.02,52.02)	1.000
Day 28	4 ± 0	8 ± 0	4 (−48.02,56.02)	0.880
*NS-V2 (7) VS S-V2 (7*)	**NS-V2(7) group**	**S-V2(7) group**		
Day 1	512 ± 0	128 ± 0	−384 (−395.17,−372.83)	< 0.001*
Day 3	64 ± 0	64 ± 0	−2.27e-13 (−11.17,11.17)	1.000
Day 5	32 ± 0	53.33 ± 18.48	21.33 (10.16,32.51)	< 0.001*
Day 7	42.67 ± 18.48	53.33 ± 18.48	10.67 (−0.51,21.84)	0.061
Day 14	16 ± 0	32 ± 0	16 (4.83,27.17)	0.005*
Day 21	16 ± 0	16 ± 0	−1.14e-13 (−11.17,11.17)	1.000
Day 28	8 ± 0	8 ± 0	−1.14e-13 (−11.17,11.17)	1.000

*MID* minimum inhibitory dilution, *սg* microgram, *mL* milliliter, *SD* standard deviation

*LS mean* least square mean, *CI* confident interval, *NS* non-sterile, *V2* vancomycin 2 g, *S* sterile

*V2(4)* vancomycin 2 g and 4 day storage, *V2(7)* vancomycin 2 g and 7 day storage

From Day 3 to Day 28, no statistically significant differences in MID were observed between non-sterilised and sterilised specimens across most storage durations. However, in the seven day storage subgroup, sterilised specimens demonstrated significantly higher antimicrobial activity at Day 5 (mean difference 21.33; 95% CI 10.16 to 32.51; *p* < 0.001) and Day 14 (mean difference 16; 95% CI 4.83 to 27.17; *p* = 0.005) compared with non-sterilised specimens. A similar trend was observed at Day 7, although this did not reach statistical significance (*p* = 0.061).

By Day 28, all sterilised specimens maintained antibacterial activity above the minimum inhibitory concentration for *S. aureus*, regardless of storage duration (S-V2: 4 ± 0; S-V2[4]: 8 ± 0; S-V2[7]: 8 ± 0), indicating preservation of sustained antimicrobial efficacy despite sterilisation.

### Vancomycin 4 g group

Across all storage duration subgroups (Table 2), formaldehyde gas sterilisation was associated with a reduction in early antimicrobial activity compared with non-sterilised specimens, with the timing and magnitude of this effect varying by storage duration.

**Table 2:** Minimum inhibitory dilution (MID) of vancomycin eluted from polymethylmethacrylate bone cement containing 4 g of vancomycin, comparing non-sterilised and formaldehyde gas-sterilised specimens at different storage durations over the 28-day elution period

Outcome	MID (սg/mL)		Mean difference	*P*-value
	mean ± SD or	mean ± SD or	(95%CI)	
*NS-V4 VS S-V4*	LS mean (95%CI)	LS mean (95%CI)		
Day 1	1024 ± 0	1024 ± 0	−6.82—13 (−13.51,13.51)	1.000
Day 3	128 ± 0	74.67 ± 26.13	−53.33 (−66.84,−39.83)	< 0.001*
Day 5	96 ± 35.05	48 ± 17.53	−48 (−61.51,−34.49)	< 0.001*
Day 7	64 ± 0	32 ± 0	−32 (−45.51,−18.49)	< 0.001*
Day 14	32 ± 0	21.33 ± 8.26	−10.67 (−24.17,2.84)	0.122
Day 21	24 ± 8.76	14.67 ± 3.27	−9.33 (−22.84,4.17)	0.176
Day 28	16 ± 0	12 ± 4.38	−4 (−17.51,9.51)	0.562
*NS-V4 (4) VS S-V4 (4*)	**NS-V4 (4) group**	**S-V4(4) group**		
Day 1	1024 ± 0	213.33 ± 73.90	−810.67 (−847.31,−774.03)	< 0.001*
Day 3	213.33 ± 73.90	128 ± 0	−85.33 (−121.97,−48.69)	< 0.001*
Day 5	64 ± 0	64 ± 0	−2.27e-13 (−36.64,36.64)	1.000
Day 7	64 ± 0	64 ± 0	−5.68—13 (−36.64,36.64)	1.000
Day 14	64 ± 0	64 ± 0	−5.68—13 (−36.64,36.64)	1.000
Day 21	32 ± 0	26.67 ± 9.23	−5.33 (−41.97,31.31)	0.775
Day 28	32 ± 0	32 ± 0	−9.09—13 (−36.64,36.64)	1.000
*NS-V4 (7) VS S-V4 (7*)	**NS-V4(7) group**	**S-V4(7) group**		
Day 1	1365.33 ± 591.21	213.33 ± 73.90	−1152 (−1362.25,−941.75)	< 0.001*
Day 3	170.67 ± 73.90	64 ± 0	−106.67 (−316.92,103.59)	0.320
Day 5	106.67 ± 36.95	53.33 ± 18.48	−53.33 (−263.59,156.92)	0.619
Day 7	64 ± 0	32 ± 0	−32 (−242.25,178.25)	0.765
Day 14	42.67 ± 18.48	32 ± 0	−10.67 (−220.92,199.59)	0.921
Day 21	32 ± 0	16 ± 0	−16 (−226.25,194.25)	0.881
Day 28	32 ± 0	16 ± 0	−16 (−226.25,194.25)	0.881

*MID* minimum inhibitory dilution, *սg* microgram, *mL* milliliter, *SD* standard deviation

*LS mean* least square mean, *CI* confident interval, *NS* non-sterile, *V4* vancomycin 4-g, *S* sterile

*V4(4)* vancomycin 4 g and 4 day storage, *V4(7)* vancomycin 4 g and 7 day storage

In specimens tested after one day of storage, no difference in MID was observed on Day 1 between non-sterilised and sterilised cement (1024 ± 0 vs 1024 ± 0; *p* = 1.000). However, significant reductions in MID were observed in the sterilised group during the early elution phase, specifically on Day 3 (mean difference − 53.33; 95% CI − 66.84 to − 39.83; *p* < 0.001), Day 5 (mean difference − 48.00; 95% CI − 61.51 to − 34.49; *p* < 0.001), and Day 7 (mean difference − 32.00; 95% CI − 45.51 to − 18.49; *p* < 0.001).

In the four day storage subgroup, the MID was significantly lower in sterilised specimens on Day 1 (mean difference − 810.67; 95% CI − 847.31 to − 774.03; *p* < 0.001) and Day 3 (mean difference − 85.33; 95% CI − 121.97 to − 48.69; *p* < 0.001). No statistically significant differences were observed from Day 5 onward. Similarly, in the seven day storage subgroup, a significant reduction in MIC was observed only on Day 1 (mean difference − 1152.00; 95% CI − 1362.25 to − 941.75; *p* < 0.001). No significant differences were detected at subsequent time points. The wide confidence intervals in the four day and seven day subgroups reflect their single-specimen-per-condition design, and the apparent precision of mean differences in these conditions should not be overstated.

From Day 14 to Day 28, no statistically significant differences in MID were observed between sterilised and non-sterilised specimens across all storage durations. At Day 28, MIC values in sterilised cement remained above the minimum inhibitory concentration for *Staphylococcus aureus* (1-day storage: 12 ± 4.38; 4-day storage: 32 ± 0; 7-day storage: 16 ± 0), indicating preservation of sustained antimicrobial activity despite sterilisation.

### Comparison between sterilised vancomycin 2 g and 4 g cement

**Fig. 3:**
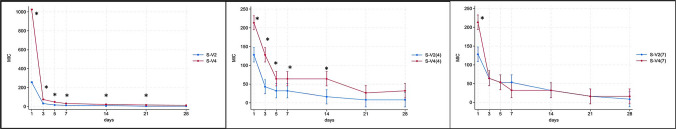
Comparison of minimum inhibitory dilution (MID) values of eluates from **sterilised** PMMA cement containing 2 g and 4 g of vancomycin against *Staphylococcus aureus* ATCC 25923 over the 28-day elution period, stratified by storage duration (1, 4, and 7 days)

Across all storage durations (Table 3), sterilised PMMA cement containing 4 g of vancomycin demonstrated consistently higher antimicrobial activity than cement containing 2 g, particularly during the early and mid-elution phases (Fig. 3).

**Table 3:** Comparison of minimum inhibitory dilution (MID) values between sterilised polymethylmethacrylate bone cement containing 2 g and 4 g of vancomycin across different storage durations over the 28-day elution period

Outcome	MID (սg/mL)		Mean difference	*P*-value
	mean ± SD or	mean ± SD or	(95%CI)	
	LS mean (95%CI)	LS mean (95%CI)		
S-v2 vs s-v4	**S-V2 group**	**S-V4 group**		
Day 1	256 ± 0	1024 ± 0	768 (758.81,777.19)	< 0.001*
Day 3	32 ± 0	74.67 ± 26.13	42.67 (33.48,51.85)	< 0.001*
Day 5	16 ± 0	48 ± 17.53	32 (22.81,41.19)	< 0.001*
Day 7	12 ± 4.38	32 ± 0	20 (10.81,29.19)	< 0.001*
Day 14	8 ± 0	21.33 ± 8.26	133.33 (4.15,22.52)	0.004*
Day 21	4 ± 0	14.67 ± 3.27	10.67 (1.48,19.85)	0.023*
Day 28	4 ± 0	12 ± 4.38	8 (−1.19,17.19)	0.088
S-v2(4) vs s-v4(4)	**S-V2(4) group**	**S-V4(4) group**		
Day 1	128 ± 0	213.33 ± 73.90	85.33 (58.54,112.13)	< 0.001*
Day 3	42.6 ± 18.48	128 ± 0	85.33 (58.54,112.13)	< 0.001*
Day 5	32 ± 0	64 ± 0	32 (5.20,58.80)	0.019*
Day 7	32 ± 0	64 ± 0	32 (5.20,58.80)	0.019*
Day 14	16 ± 0	64 ± 0	48 (21.20,74.80)	< 0.001*
Day 21	8 ± 0	26.67 ± 9.24	18.67 (−8.13,45.46)	0.172
Day 28	8 ± 0	32 ± 0	24 (−2.80,50.80)	0.079
S-v2(7) vs s-v4(7)	**S-V2(7) group**	**S-V4(7) group**		
Day 1	128 ± 0	213.33 ± 73.90	85.33 (57.21,113.46)	< 0.001*
Day 3	64 ± 0	64 ± 0	−2.84e-14 (−28.12,28.12)	1.000
Day 5	53.33 ± 18.48	53.33 ± 18.48	−1.28e-13 (−28.12,28.12)	1.000
Day 7	53.33 ± 18.48	32 ± 0	−21.33 (−49.46,6.79)	0.137
Day 14	32 ± 0	32 ± 0	−5.68e-14 (−28.12,28.12)	1.000
Day 21	16 ± 0	16 ± 0	−7.11e-14 (−28.12,28.12)	1.000
Day 28	8 ± 0	16 ± 0	8 (−20.12,36.12)	0.577

*MID* minimum inhibitory dilution, *սg* microgram, *mL* milliliter, *SD* standard deviation

*LS mean* least square mean, *CI* confident interval, *NS* non-sterile, *S* sterile, *V2* vancomycin 2 g

*V4* vancomycin 4 g, *V2(4)* vancomycin 2 g and 4 day storage, *V2(7)* vancomycin 2 g and 7 day storage, *V4(4)* vancomycin 4 g and 4 day storage, *V4(7)* vancomycin 4 g and 7 day storage

In specimens tested after one day of storage, MID values were significantly higher in the S-V4 group compared with the S-V2 group from Day 1 through Day 21 (*p* < 0.05 for all), with the greatest difference observed on Day 1 (1024 ± 0 vs 256 ± 0; mean difference 768; 95% CI 758.81 to 777.19; *p* < 0.001). This difference diminished by Day 28 and did not reach statistical significance (*p* = 0.088).

In the four day storage subgroup, S-V4(4) demonstrated significantly higher MID values than S-V2(4) from Day 1 through Day 14 (*p* ≤ 0.019), indicating superior antimicrobial activity during both the burst and early diffusion phases. No statistically significant differences were observed at Days 21 and 28.

In the seven day storage subgroup, S-V4(7) exhibited a significantly higher MID than S-V2(7) on Day 1 (*p* < 0.001). However, no significant differences were observed between groups at subsequent time points from Day 3 through Day 28.

## Discussion

Periprosthetic joint infection (PJI) and chronic osteomyelitis remain among the most challenging conditions in orthopaedic practice and often require surgical debridement combined with prolonged local antimicrobial therapy [10, 11]. Antibiotic-loaded polymethylmethacrylate (PMMA) cement is widely used as a spacer in two-stage revision arthroplasty and as a local antibiotic delivery vehicle in osteomyelitis, providing high local antibiotic concentrations while minimizing systemic toxicity [12]. In current practice, antibiotic-loaded cement spacers are most commonly prepared intraoperatively, a process that typically requires approximately a total of 15–30 min and prolongs operative time [13, 14]. Although commercially prefabricated antibiotic cement spacers are available, their clinical adoption is limited by higher cost, restricted antibiotic selection, and reduced flexibility in antibiotic dosing [14, 15]. Prefabrication and sterilisation of custom antibiotic-loaded cement spacers prior to surgery may therefore reduce operative duration and surgical morbidity while allowing greater customisation; however, concerns remain regarding the effects of sterilisation and subsequent storage on antibiotic activity. Accordingly, this study evaluated the impact of formaldehyde gas sterilisation and storage duration on the antimicrobial activity of vancomycin-loaded bone cement using minimum inhibitory concentration analysis against *Staphylococcus aureus*.

The present study used a functional bioassay approach (MID testing) to assess the impact of formaldehyde sterilisation and storage duration on the antimicrobial efficacy of vancomycin-loaded bone cement against *S. aureus*. Our findings demonstrate that sterilisation primarily reduces antimicrobial activity during the early elution phase (Days 1–7), while sustained-phase elution (Days 14–28) remains comparable between sterilised and non-sterilised specimens across all storage durations. One possible explanation for this observation is that formaldehyde modifies surface-accessible vancomycin while sparing embedded antibiotic. This hypothesis is supported by the work of Heck et al*.*, who demonstrated that formaldehyde exposure can chemically modify vancomycin at exposed molecular sites, preferentially reducing the activity of surface-accessible antibiotic while sparing protected fractions [16]. When applied to PMMA cement, this mechanism would predict selective inactivation of surface-associated vancomycin during sterilisation, with preservation of the bulk antibiotic reservoir that elutes during the sustained phase. However, our study did not directly measure vancomycin chemical modification or surface-versus-bulk antibiotic distribution, and this interpretation remains speculative.

Consistent with our findings, Shaw et al. reported that antibiotic-loaded PMMA beads retained antimicrobial activity after sterilisation and storage for up to six months, comparable to freshly sterilised specimens, with no observed decline attributable to storage duration [9]. Although different sterilisation methods were evaluated, their results support the concept that sterilisation and storage do not compromise sustained antimicrobial activity of antibiotic-loaded PMMA cement, provided that the bulk antibiotic reservoir remains intact.

Based on current CLSI antimicrobial susceptibility criteria, vancomycin MIC values ≤ 2 µg/mL are considered inhibitory for *Staphylococcus aureus* [17]. In this study, antimicrobial potency of all sterilised vancomycin-loaded cement groups remained several-fold above this threshold throughout the 28-day elution period, indicating preservation of clinically relevant antimicrobial activity. Importantly, eradication of bacterial biofilms, especially in PJI, requires higher (10—100 times) antibiotic concentration than planktonic MIC values, which are generally considered necessary for effective bacterial eradication [18–20]. During the early and mid-elution phases in this study, antimicrobial potency values fell within or approached this biofilm-effective range., especially in 4—g vancomycin group.

Although longer storage durations (4 and 7 days) were associated with a significant reduction in early burst antimicrobial activity in the 4—g group, particularly on Day 1, all sterilised specimens maintained antimicrobial potency above inhibitory thresholds during the sustained elution phase, with no significant differences at later time points (Days 21–28). These preliminary findings suggest that prefabricated PMMA cement containing 4 g of vancomycin may retain adequate sustained antimicrobial activity after storage of up to seven days following formaldehyde sterilisation. However, the four day and seven day storage conditions each comprised a single sterilised specimen, which substantially limits statistical power and precludes definitive conclusions about the effect of storage duration. These observations should be considered hypothesis-generating, and confirmation in larger, well-powered studies is essential before any clinical recommendations can be made. Furthermore, because this was an in vitro study, translation to clinical outcomes requires additional validation, including direct antibiotic quantification and in vivo pharmacokinetic modelling.

This study has several limitations that should be acknowledged. First, the number of specimens per subgroup was small, and this constraint has direct implications for the interpretation of statistical comparisons. In the one day storage conditions, two replicate specimens per condition (sterilised and non-sterilised) provided minimal but some degree of replication. In contrast, the four day and seven day storage subgroups each contained only a single sterilised specimen; within these conditions, no within-group variance could be estimated, confidence intervals reflect model-estimated uncertainty rather than observed replication, and the apparent statistical significance of between-group differences should be interpreted with considerable caution. All subgroup comparisons should be considered exploratory and hypothesis-generating. Larger studies with pre-specified sample sizes are required to confirm these findings.

Second, antimicrobial activity was assessed using a functional bioassay against a single reference strain (*S. aureus* ATCC 25923). The PJI pathogen spectrum is considerably broader: coagulase-negative staphylococci, such as *S. epidermidis*, are the most frequently isolated organisms, accounting for approximately 37% of PJI episodes, followed by *S. aureus* at approximately 25%, with polymicrobial infections comprising a further 9–25% of cases depending on the institution and case mix [21, 22]. Gram-negative organisms and anaerobes contribute an additional proportion, particularly in early postoperative infections. The extent to which the MID findings for vancomycin observed against *S. aureus* ATCC 25923 are applicable to coagulase-negative staphylococci, methicillin-resistant isolates, or Gram-negative pathogens cannot be inferred from the present data. Future work should incorporate testing against *S. epidermidis*, MRSA clinical isolates, and representative Gram-negative organisms to broaden the clinical relevance of the findings.

Third, although MID testing provides a standardised functional measure of antimicrobial potency in the eluate, it does not capture the complex interactions between antibiotic-loaded cement and biofilm-associated bacteria in vivo. The minimum biofilm eradication concentration (MBEC) for staphylococci in PJI has been shown to exceed the planktonic MIC by more than 8000-fold [23], and the relationship between MID values and biofilm eradication capacity was not assessed in this study. Direct vancomycin quantification was not performed; therefore, elution kinetics and the dose–response relationship between antibiotic concentration and antimicrobial potency could not be fully characterised. Future studies should incorporate direct quantification, for example, by high-performance liquid chromatography, to provide complementary pharmacokinetic data alongside functional bioassay results, as has been done in other PMMA elution studies [8].

Fourth, only formaldehyde gas sterilisation was evaluated in this study. Other commonly used sterilisation modalities, including autoclaving, ethylene oxide, and gamma irradiation, may produce different effects on antibiotic stability and elution characteristics. Shaw et al. demonstrated preserved bioactivity of vancomycin-tobramycin PMMA beads across autoclave, ethylene oxide, and UV sterilisation methods [9]. Whether the pattern observed with formaldehyde gas is specific to chemical modification by formaldehyde, or would be reproduced with thermal or radiation-based methods, remains an important open question, particularly for institutions without access to formaldehyde gas sterilisation units.

Finally, this was an in vitro study only, and clinical factors such as local tissue perfusion, host immune response, mechanical loading of cement spacers, and protein binding of vancomycin in synovial fluid were not assessed. Available in vivo data suggest that intra-articular antibiotic concentrations decline rapidly after implantation, falling below the planktonic MIC for many organisms within 24 h in some series [24], underscoring the importance of caution in extrapolating in vitro elution profiles to clinical outcomes. Accordingly, extrapolation of these findings to clinical practice should be undertaken with caution, and prospective in vivo or clinical studies are required to confirm whether the antimicrobial activity observed in vitro translates to infection eradication in patients.

## Conclusion

Formaldehyde gas sterilisation of vancomycin-loaded PMMA cement transiently reduces early burst antimicrobial activity in vitro but does not compromise sustained antibacterial efficacy over 28 days. Higher vancomycin loading (4 g) mitigates the impact of sterilisation and storage, providing more robust antimicrobial activity than 2—g cement across all storage durations tested. These preliminary in vitro findings suggest that sterilised PMMA cement containing 4 g of vancomycin may maintain MID values above inhibitory thresholds for *S. aureus* for up to seven days of post-sterilisation storage, supporting further investigation of prefabricated spacers as a strategy to reduce operative time. Clinical translation, however, requires confirmation in larger studies incorporating direct antibiotic quantification, testing against a broader panel of clinically relevant pathogens, and prospective in vivo validation.

## Data Availability

No datasets were generated or analysed during the current study.
